# Sex Workers Perspectives on Strategies to Reduce Sexual Exploitation and HIV Risk: A Qualitative Study in Tijuana, Mexico

**DOI:** 10.1371/journal.pone.0072982

**Published:** 2013-08-30

**Authors:** Shira M. Goldenberg, David Engstrom, Maria Luisa Rolon, Jay G. Silverman, Steffanie A. Strathdee

**Affiliations:** 1 BC Centre for Excellence in HIV/AIDS, Division of AIDS, Department of Medicine, University of British Columbia, Vancouver, British Columbia, Canada; 2 Department of Medicine, Division of Global Public Health, University of California San Diego, La Jolla, California, United States of America; 3 School of Social Work, San Diego State University, San Diego, California, United States of America; 4 School of Medicine, Universidad Xochicalco, Tijuana, Baja California, Mexico; Tulane University, United States of America

## Abstract

Globally, female sex workers are a population at greatly elevated risk of HIV infection, and the reasons for and context of sex industry involvement have key implications for HIV risk and prevention. Evidence suggests that experiences of sexual exploitation (i.e., forced/coerced sex exchange) contribute to health-related harms. However, public health interventions that address HIV vulnerability and sexual exploitation are lacking. Therefore, the objective of this study was to elicit recommendations for interventions to prevent sexual exploitation and reduce HIV risk from current female sex workers with a history of sexual exploitation or youth sex work. From 2010–2011, we conducted in-depth interviews with sex workers (n = 31) in Tijuana, Mexico who reported having previously experienced sexual exploitation or youth sex work. Participants recommended that interventions aim to (1) reduce susceptibility to sexual exploitation by providing social support and peer-based education; (2) mitigate harms by improving access to HIV prevention resources and psychological support, and reducing gender-based violence; and (3) provide opportunities to exit the sex industry via vocational supports and improved access to effective drug treatment. Structural interventions incorporating these strategies are recommended to reduce susceptibility to sexual exploitation and enhance capacities to prevent HIV infection among marginalized women and girls in Mexico and across international settings.

## Introduction

Globally, women and girls in the sex industry experience disproportionately high risk of HIV infection [1], and the reasons for and context of sex industry involvement have key implications for HIV risk and prevention [2]–[7]. Evidence suggests that sexual exploitation and youth involvement in sex work confer serious health-related harms among marginalized women and girls. In this study, we define sexual exploitation as forced/coerced sex exchange, and youth sex work as the exchange of sex prior to age 18. These are considered to be the primary components of sex trafficking as defined by the United Nations Palermo Protocol [8]. Although population-based estimates of the prevalence of sexual exploitation and youth sex work are difficult to obtain due to their hidden nature [9], epidemiological studies indicate that up to 40% of female sex workers and marginalized adolescents are involved in sex work as youth [10]–[12], and between 10–25% may be forced or deceived into the sex industry [13], [14].

Previous studies indicate that sexual exploitation and youth sex work confer greatly heightened vulnerability to sexual and physical violence, sexually transmitted infections (STIs), and HIV infection [14]–[17]. For instance, in eastern India, almost 25% of sex workers participating in an epidemiological study reported that they had been forced or deceived to exchange sex; these participants were more likely to report violence when they began to exchange sex, which was associated with higher odds of HIV infection [13]. In a national sample of female sex workers in Thailand, trafficked women were more likely to report sexual violence at initiation into the sex industry, workplace violence or abuse, and condom failure or non-use [14].

Despite the elevated HIV vulnerabilities faced by this population, appropriate evidence-based public health interventions are lacking. Whereas numerous promising behavioral and structural interventions have been developed for female sex workers [18]–[21], interventions for females involuntarily involved in the sex industry or who begin sex work as youth have yet to be developed [11]. To begin building such interventions, there is a need to solicit the perspectives of women and girls who have experienced these circumstances [22], [23]. Although a substantial volume of literature exists documenting the needs of trafficked persons [23]–[38], very few studies have given voice to the perspectives of those who have survived such abuses [22]. Therefore, this study aimed to explore potential strategies to reduce vulnerability to HIV and sexual exploitation by gathering the opinions and perspectives of women with a history of sexual exploitation or youth sex work.

Latin America is reported to be the source of hundreds of thousands of persons trafficked across international borders annually, yet it is one of the most under-researched and under-funded regions on issues of sexual exploitation [28]. Mexico is a large source, transit, and destination country for trafficked persons [39]. In Mexico, complex social and economic factors, including those related to migration, human rights abuses, poverty, gender inequalities, and organized crime, are believed to contribute to vulnerability to exploitation and adolescent sex work [37], [38], [40], [41]. Women, undocumented migrants, and young people have been reported as populations most vulnerable to sexual exploitation in Mexico [38], [40], [41]. In spite of these elevated risks, research aimed at elucidating an effective response, such as measures related to victim assistance and public policy approaches, remains limited in Mexico [37], [38].

The border between Tijuana, Mexico and San Diego, USA, is the world's busiest international land crossing [42], and is a key location for the repatriation of undocumented Mexican migrants from the U.S. Sexual exploitation and adolescent sex work are believed to be rife in Tijuana, largely due to the risks posed by the city's overlapping sex and drug trades and intense population mobility. Large numbers of women and children from Central America and southern and central Mexico are reportedly trafficked to Mexican sex tourism locations such as border areas, tourist destinations, ports, and areas hosting migrant workers [34]. Child sex tourism is often found in Mexican border and tourist areas, including Tijuana [39], where media reports have recently drawn attention to numerous cases of child sexual exploitation [43], [44].

Recently, our qualitative research in Tijuana linked vulnerability to forced sex exchange, youth sex work, and HIV to prior experiences of gender-based violence, drug use, economic marginalization, coercive migration, and deportation from the United States [3], [45]. Informed by these findings and participants' narratives, the aim of this study was to gather suggestions for ways to reduce vulnerability to HIV and sexual exploitation from the perspectives of sex workers with a history of sexual exploitation or youth sex work in Tijuana, Mexico.

## Methods

### Ethics statement

The study protocol was approved by IRBs at the University of California, San Diego (UCSD) and El Colegio de la Frontera Norte in Tijuana, Mexico (COLEF). All procedures were conducted in accordance with the ethical standards expressed in the Declaration of Helsinki.

Study interviewers received training according to WHO guidelines for research with trafficked women [46] as well as in relevant ethical and methodological procedures (e.g., protection of participant confidentiality, anonymity, and safety; qualitative interviewing). All potential interviewees were explained the purpose of the study, the voluntary nature of participation, and its risks and benefits, and provided written informed consent prior to participating in the study.

### Setting

Tijuana is a popular sex tourism destination. The city hosts large sex work and injection drug use scenes, which are largely concentrated in the *Zona Norte*, a red light district in which thousands of sex workers sell or trade sex to clients from the U.S., Mexico, and international locations [47]–[49]. Sex work in the *Zona Norte* is quasi-legal; to avoid persecution by police, adult sex workers are required to undergo routine STI/HIV testing to maintain health permits, which are unavailable to minors.

Tijuana is also experiencing an emerging HIV epidemic, and women and youth in sex work are among the most affected populations; HIV prevalence has increased from <1% to 6% among female sex workers in Mexico-U.S. border cities in the past decade [50], and is >12% among those who also inject drugs [51].

### Data collection

From November 2010 to July 2011, we conducted in-depth qualitative interviews with 31 sex workers who reported a history of prior sexual exploitation or youth sex work. Data collection included 31 initial interviews and 6 follow-up *member-checking* interviews with a sub-group of these women (37 interviews in total). Participants were recruited from a larger study among 420 female sex workers and their male partners in Tijuana and Ciudad Juarez (*Proyecto Parejas*; PI: Strathdee). Data collection for *Projecto Parejas* included quantitative surveys and biological HIV/STI testing among female sex workers, as previously described [52]. Women were recruited by targeted sampling by outreach workers in areas where sex work and drug use commonly occur (e.g., street, bars).

Eligible women for *Proyecto Parejas* were ≥18 years old, traded sex in the past month, reported lifetime use of heroin, cocaine, crack, or methamphetamine, had a stable partner for at least 6 months, had sex with that partner in the last month, and were willing to recruit their partner for the study. From this sampling frame, we conducted interviews with Tijuana-based women who were (1) <18 years old the first time they first traded sex for money, drugs, shelter or other resources; (2) forced, coerced, or deceived into trading sex (e.g., sold/traded, kidnapped, forced, or tricked to exchange sex); and/or (3) transported and forced to exchange sex against their will (e.g., moved between cities as a sex worker and forced/coerced to exchange sex). These measures were assessed during the *Projecto Parejas* questionnaire; we subsequently developed a purposive sample [53] of women whose survey responses met one or more of these criteria and who represented a range in age, nationality (e.g., Mexican vs. foreign-born), and reasons/contexts for beginning to exchange sex (e.g., adolescent entry, forced). Women who met one or more of these criteria were invited to participate in a qualitative interview.

In-depth interviews were conducted in private offices by female interviewers, including a PhD student (SG), a medical student (MR), as well as two outreach workers employed by a HIV prevention organization in Tijuana. All members of our team had previous experience working on HIV prevention studies with sex workers, their clients, and/or people who use drugs in Tijuana, and these longstanding relationships with our study population facilitated rapport between participants and interviewers.

Interviews were conducted in the participants' preferred language (Spanish or English), audiotaped, and lasted approximately 1.5 hours. Interviews were informed by ethnographic methods [54] and loosely followed an open-ended guide, which was pilot tested and iteratively revised throughout the course of data collection and analysis. Interview questions elicited information regarding the circumstances surrounding participants' entry into the sex industry, migration history, perceived HIV/STI risk, drug use patterns, access to health and social services, and suggestions for prevention and care. Follow-up *member-checking* interviews were conducted with a sub-group of previously interviewed women (n = 6), which provided opportunities to gather their feedback on our preliminary findings, explore salient themes in greater depth, and further elicit women's suggestions for interventions to reduce vulnerability to HIV and sexual exploitation. All women received condoms, HIV prevention information, and $20 USD, and were offered referrals to health and social services.

### Data analysis

All interviews were transcribed verbatim and accuracy checked by SG. All personal identifiers were removed and replaced by unique pseudonyms to protect participants' identities. Data were coded and managed using the software NVivo 9.0 (QSR, AUS). The analysis was led by SG in collaboration with co-authors, who provided input regarding the identification and interpretation of themes. Analyses were restricted to 30 women who began sex work 15 years ago on average (range 4 to 33); one participant was excluded from the analysis, whose sex industry entry occurred over 40 years prior and reflected a sufficiently different historical context to warrant exclusion. Analyses employed the constant comparative method to describe the content and structure of the data [55]. We began with open coding to inductively generate an initial coding scheme derived from participants' language and experiences. We continued to group and regroup codes until the analysis yielded a comprehensive set of themes that described women's main suggestions for interventions to prevent sexual exploitation and HIV infection.

## Results

### Participant characteristics

Women's median age was 33 (range: 19–54) and they had a median of 6 years of education (Table 1). Drug use was highly prevalent, since eligibility criteria included a history of drug use. By design, 25 women began to exchange sex as youth, 11 were forced/coerced to exchange sex, and two were transported and forced to exchange sex; as many participants experienced more than one of these experiences, these categories are not exclusive. The median age of sex industry entry was 16 (range: 12–28), and 11 women reported a history of rape (median age: 14). Five participants (17%) tested positive for an STI or HIV infection (Table 1).

**Table 1:** Participant characteristics (N = 30), 2010–2011.

Variable	(n = 30)
Age, *years* a	33 (19–54)
Education, *years* a	6 (1–15)
Birthplace	
*Tijuana, Mexico*	11 (36.7%)
*Other Mexican city*	17 (57.0%)
*United States*	1 (3.3%)
*Central America*	1 (3.3%)
Began to exchange sex <18 years old*	25 (85.3%)
Forced, deceived, or coerced to exchange sex*	11 (36.7%)
Transported and forced to exchange sex*	2 (6.67%)
Age when first traded sex, *years* a	16 (12–28)
History of violence*	
*Ever experienced a traumatic event*	9 (30.0%)
*Ever raped*	11 (36.7%)
*Ever physically abused*	7 (23.3%)
Positive for any STI/HIV	5 (16.7%)
Drugs used, *past 6 months* *	
*Heroin*	19 (63.3%)
*Cocaine*	6 (20.7%)
*Methamphetamine*	22 (75.9%)
Injected drugs, *past 6 months*	18 (60.0%)

a Median (range).

* Categories not exclusive.

NOTE: Data are N (%) of women, unless otherwise indicated.

### Findings

Women's recommendations included efforts to *(1) reduce susceptibility to exploitation* by providing social support and employing peer-based approaches; *(2) mitigate harms* by increasing access to HIV prevention, strengthening the response to gender-based violence, and providing psychological support for survivors of violence; and *(3) provide opportunities to exit the sex industry* through vocational trainings/placements and effective drug treatment.

### (1) Reduce susceptibility to exploitation

***Provide social support:*** The narratives of many participants in this study framed their histories of early or forced exchange as a result of the limited options they experienced as homeless youth or recent migrants to Tijuana. Reflecting upon this, women indicated the need for shelter and related social support to reduce vulnerability to sexual exploitation among migrants and vulnerable youth. As one woman described her perception of the risks posed by Tijuana's *Zona Norte*, especially for recent migrants:

They don't realize that there are people who live here off of catching the people who come, if it's not to steal from them, to convince them [to do sex work]…Unfortunately, Tijuana has a lot of business through other people.

[Melissa, Age 32]

In light of these risks, participants frequently discussed migrant and young women's needs for social supports, especially deportees. For example, one deportee explained that she was deported from the U.S. (after living there for almost her entire life) without any economic resources or kinship ties that she could rely upon for support. Not having been offered shelter, food, or other support to assist her transition to life in Mexico, she reflected on how provision of such resources could have assisted her and others facing similar situations:

The best thing I would just say is to link or provide them with a place to stay…Something for women only, as soon as they came across [the border], a program that could help them with a place to stay, even in exchange for work…There needs to be a home especially for the Chicano [Mexican American] type of person and girls like myself that come out here…you're not used to the way of how things go down here…because us girls with low options, there's a lot of people that prey on girls like us.

[Gloria, Age 29]

Other women shared similar sentiments. As one participant stated the need for services to increase awareness and reduce the health and social vulnerabilities of recent migrants:

They [migrants] risk their lives when they come…there should be something that would give you a chance to show up and know exactly what you're getting into…so they wouldn't have to come and be dealing with all kinds of stuff. They're hungry, cold, you know - some sort of service to help us take it easy.

[Esperanza, Age 25]

***Improve awareness through peer education:*** Women discussed the need to increase awareness of sexual exploitation as a means of preventing abuse against marginalized women and girls. Participants often described the ways that their own experiences could help raise awareness of sexual exploitation in their community. In one case, a participant who had been disproportionately exposed to prior violence and exploitation, including sexual violence while being smuggled from Guatemala to Los Angeles, as well as forced sex exchange in Tijuana, often acted as a resource to her peers. She shared her perception of the importance of sharing stories like hers for drawing attention to issues of sexual exploitation:

It's very important, for me and for other people…to realize, to open their eyes and see the reality, that this is happening around us…There are some [sex workers] who come and start asking or telling me what happens to them…the girls who work with these people [traffickers], well there's authority, they have to deal with it, there are some who come back all beat up, that they did this and that to them.

[Perla, Age 34]

Peer education was a frequently suggested means of increasing awareness of sexual exploitation among adolescents, migrants, and others women and girls who had recently entered the sex industry. Peers were often depicted as the most trusted group with whom participants would be able to identify, highlighting the importance of grounding interventions in peers' experiences and potential for peer-delivered interventions:

You would need someone that has the same background as us so that they can better understand the situation we're in. That way they'd be able to help us, not make things worse for us. Because a lot of times instead of helping you, they turn you in - that's why we don't talk.

[Esmeralda, Age 38]

If people on the street try to take advantage of me…especially in a new city…I'd be scared, especially with all the stuff you hear in the papers about Tijuana…But someone like me, I've got the tattoos and they're going to see that I'm real and I've really been through what I'm telling you…like, ‘I could talk to this girl because she does understand me, she's been through it…maybe I could learn something from her.’

[Gloria, Age 29]

Women often discussed the importance of sharing the lessons they learned during their early years in the sex industry. Some described instances in which they had provided advice to peers (e.g., avoid migrating alone; don't trust strangers), while many others expressed interest in doing so:

Maybe I could, if there's a girl like me, [advise] for her to not…end up like I did.

[Esperanza, Age 25]

I'd love to go out there and speak to girls…because its messed up out here and it's scary…you feel like nobody wants to hear it…[it] would prevent them from having to go through a lot of the crap that you never imagine would be this painful.

[Gloria, Age 29]

### (2) Mitigate harms

***Enhance access to HIV prevention resources:*** Participants often cited conventional HIV prevention and care services as unavailable or inaccessible, highlighting the need to increase access to reduce the harms they faced. Free or low-cost male and female condoms, harm reduction messaging, and HIV/STI testing were cited as pillars of HIV promotion they desired improved access to. As one participant explained the importance of being aware of the different modes of HIV transmission and their prevention:

There are many [sex workers] who don't know all of the risks [HIV]…and they get with whoever, for a little bit of money sometimes, with guys who come from the other side [the United States]. Or many don't know that they need to be careful to not use the same needle, or that they need to change the cooker.

[Angelica, Age 44]

Geographic, financial, and institutional factors such as the time, travel, and resources required to access HIV-related services were common barriers to access. Efforts to reduce these barriers were perceived as essential to bolster HIV/STI prevention capacities:

I would like there to be more places that you could go for [HIV] testing. For people that live far away, so that people could just stop by and get tested. Or they could give out condoms or a presentation.

[Jimena, Age 34]

They do help you at the health center but you say, ‘no I won't go…because they're going to charge me here.’

[Alejandra, Age 24]

In addition to conventional sources of health information and care, peers were described as key sources of HIV-related information, highlighting the potential for peer-based HIV prevention efforts in conjunction with increasing awareness of exploitation (see *Improve awareness through peer education*). As one woman described her efforts to inform her peers about the importance of consistent condom use:

I help them…open their eyes so that they don't get blinded. Sometimes it's as if they don't care about their lives. There are some girls who are, wow, they're just young girls…I give them the advice that's given to me…they mention it [HIV] a lot, they ask for my opinion and I have to advise them…I tell them, ‘Look, because of drugs, you forget that you have to use one [a condom]…Always use it, because tomorrow when you're sick, who's going to look after you?’

[Perla, Age 34]

When asked about any advice they might give a young woman today, many participants shared HIV prevention tips to promote condom use, which included always carrying condoms, using female-controlled methods such as female condoms, and avoiding drug and alcohol use during sexual transactions:

‘Be careful’…if somebody doesn't want to use a condom, there's a reason.

[Melissa, Age 32]

Make sure you are always prepared…whether you're planning to go to work or not, carry condoms…That is the best thing to do.

[Gloria, Age 29]

In addition to peer-delivered messages, participants suggested that assistance in overcoming institutional barriers to health services would enhance their capacity to mitigate HIV risk and access care. Participants often reported difficulties obtaining government-issued identification such as a birth certificate, which are required to register for and access government-funded healthcare in Mexico. Migrants were especially likely to report such challenges as barriers to accessing health and social services upon their arrival to Tijuana. As one foreign-born, HIV-positive migrant put it,

Like myself, there are many people who don't have identification, who don't have a birth certificate. A lot of people I know with HIV are in that situation, they can't get the treatment, they can't get the tests…it's not accessible.

[Melissa, Age 32]

***Strengthen the response to gender-based violence:*** Gender-based violence was common among participants, who frequently recounted histories of childhood abuse and alarming levels of violence related to their entry into the sex industry. Occupational and intimate partner violence were also persistent in their current lives, leading them to characterize sex work as inherently dangerous. As one participant stated, “It's difficult. Hoping that they [clients] won't hit you…it's dangerous, you're risking yourself being there.”

To mitigate such harms, participants expressed the need to strengthen broader responses to gender-based violence. For instance, to address intersecting experiences of exploitation and violence, one sex worker discussed the need to rely on existing legal frameworks:

The people who have their bodyguards, their pimps…they have to be paying, turn it in…If they don't turn it in they're beaten, and well that's not fair, that's what laws are for. For them to press charges and put a stop to this, because it's just going to be worse…it's better to put a stop to it.

[Perla, Age 34]

However, most women felt powerless to seek justice and prosecute perpetrators of violence and exploitation. Corruption, impunity, and fear of retribution or further criminalization were common barriers to reporting crimes:

If the guy has a lot of money, he can give a different version and they'll put me in jail. I'd rather stay quiet.

[Juliana, Age 36]

A lot of girls, the mistake they make is that they go with the judge…they shouldn't talk…I know that it'll be worse for me, so I leave it alone…This lady one time, she pressed charges; she didn't even last three days. They found her…they beat her to death, that's why it's better if you stay quiet.

[Esmeralda, Age 38]

I was scared because they said that they would do things to me and there were a lot of men [involved]. I thought that they might do something to me if I told anyone…I was scared.

[Marisol, Age 34]

The case of a participant who had sought police assistance after escaping from a kidnapper after a month of being held hostage is illustrative. This woman found a sympathetic taxi driver who took her to the police station, where she was unable to report the kidnapping due to stigma and gender-based discrimination:

They're [the police] supposed to be there to help, not to turn their back on someone. They're supposed to be there to support us…they didn't want to believe me and well to be honest, that's not fair.

[Perla, Age 34]

Participants often perceived institutional reforms to protect and assist survivors of violence and exploitation as necessary to encourage reporting of such crimes. As one participant shared her perspective:

It would be better to implement other political laws…They could establish a center that can give you protection as soon as you press charges against someone.

[Esmeralda, Age 38]

In many cases, violence was directly committed against participants by police officers, leading to deep-rooted mistrust and skepticism regarding the usefulness of reporting violence to law enforcement. The following quote illustrates the types of police violence, extortion, and harassment that were commonly reported by participants:

Sometimes they [the police] want oral sex or they take your money…you feel really used, laughed at…you say, ‘this jerk took advantage of his patrol car, his uniform and everything, of power.’ Yeah, in that sense I'm also afraid of the police.

[Angelica, Age 44]

***Provide psychological support:*** Women's narratives suggested that psychological trauma inhibited their capacities to prioritize and respond to HIV risk. Often aware of this, participating women frequently discussed the need for psychological support to promote their wellbeing and enhance their ability to engage in HIV prevention. Many indicated that they had not recovered from past instances of abuse, including sex trafficking and childhood abuse, both of which were linked to future susceptibility to violence and HIV:

I have so many things that I want to talk about…There's a lot of things relating to addictions, family, or emotions that happen in life that you don't have the guts to tell people, that you will only tell a professional…Someone that you know won't criticize you, because most of all, you fear other's peoples' opinions about you.

[Marisol, Age 34]

Women felt that increasing access to non-judgmental counseling was needed to help them cope with their past, especially related to childhood abuse, addictions, and their early years in the sex industry:

Those that are trying to escape from something should go somewhere they could help them and could give them psychological therapy. There aren't any places like that here…well, where they understand you.

[Selena, Age 22]

We need someone to talk to…because like what happened with me, I cried, I felt bad, and I had to go out [to exchange sex]…when it first happened, I felt horrible, nothing could make me feel better…that's why I'm saying that we need someone to talk to, mainly.

[Esperanza, Age 25]

Interestingly, women also spoke of their participation in qualitative interviews as addressing some of these needs, as they had provided a rare opportunity for them to discuss their past experiences with a trusted, non-judgmental listener:

The only thing that always got me to move forward has been through these interviews…to share one's experience…only then can other people know what they're going into.

[Melissa, Age 32]

It helps us…[to] know that there are people who [emotionally] support me…You're going to help a lot of people, a lot of women, because there are many who don't talk about everything and they need to clear their mind.

[Viviana, Age 34]

### (3) Provide opportunities to exit the sex industry

***Vocational trainings and placements:*** In contrast to their initial entry into the sex industry due to coercion or during adolescence, most participants situated their current engagement in adult sex work as a response to poverty, family obligations, and limited alternatives. Although women some related their choice to remain in sex work to the flexible schedule and higher earnings offered by sex industry work, others emphasized the importance of making broader career options available to those who wished to exit sex work. Some sex workers voiced their desire to pursue alternative employment opportunities, recommending job placements and trainings as central to support these aspirations:

The first thing that I would like is…a good and stable job so I could leave this…where I could clean houses or at a restaurant, anything…I would dedicate myself to my new job and I would leave all this, so I could stop having to deal with people that I don't even know.

[Perla, Age 34]

Q: What would you like for there to be at the service providers…to meet your needs?

A: There are jobs…but well, no, one doesn't have the means to study, and many don't, not just me…many would take a computer course, or beauty [course], to look for a better job. There are a lot that are happy with what they do, but there are a lot who aren't, who do it because we don't have another way of getting a job.

Q: If you had that opportunity, would you take it…?

A: Of course I would.

[Melissa, Age 32]

Some women also emphasized the importance of furthering their education as a means of improving their future employment opportunities:

I want to start going to school, I don't know how to read very well, [and] I don't know how to write…because I don't want to continue being a prostitute, I want to have a career.

[Luisa, Age 40]

**Improve access to drug treatment:** Many women identified drug use as a powerful barrier to reducing their sex work dependence. They often discussed the need for effective drug treatment to promote their overall wellbeing, linking drug treatment to their goals of leaving the sex industry:

You have to get away from this lifestyle, for one who is a drug addict, there's never going to be a way out…you have to get away from this so that you can change.

[Alejandra, Age 24]

I'm worth a lot and if I set my mind to something I can do it, no matter what it is. But first, I have to be clean, I have to be okay in order to do what I have to do.

[Viviana, Age 34]

Despite their efforts to access treatment, many women described failed efforts to abstain from drug use. Most recounted mistreatment at drug treatment facilities, which was described as including forced labor, physical abuse, stigmatization, and sexual violence. For example, one participant was raped by the director of a treatment center shortly after her arrival, and explained that such abuses were a common occurrence among sex workers referred to drug treatment centers. As the following quotes illustrate, such experiences posed powerful barriers to drug treatment access:

At a [treatment] center, they call you a whore here and they say the same thing over there, so I think, why the hell would I go then? For them to call me a whore?

[Luisa, Age 40]

While I was in the rehabilitation center I was beat up, they treat you very badly…the rehabilitation center owned pigs and they'd make us clean them as a punishment. They'd make us take showers with the same water that they used to clean their food…we're not animals, and it's not right.

[Viviana, Age 34]

Women also expressed concerns regarding the lack of counseling or other supports within such programs to address more upstream factors that they perceived to be driving their substance use. Their narratives suggest that drug treatment, when unaccompanied by appropriate psychological support, was ultimately insufficient to treat deep-rooted addictions:

We have rehabilitation centers…they talk about drugs and everything, but they never talk about the problems that we have. Those problems make us want to take drugs or get drunk, well, so we don't feel anything…[We need] a place where they offer those services.

[Selena, Age 22]

In addition to the need for treatment centers to respect their dignity and provide counseling, methadone therapy was as a desired substance use intervention; however, opioid substitution therapy (OST) (e.g., methadone) was described as costly and difficult to maintain while remaining in Tijuana's *Zona Norte*, where heroin is widely available. Treatment facilities were described as typically relying on abstinence-based treatment, limiting their potential effectiveness among women caught in the grips of addiction:

Q: So, what's motivating you to keep doing sex work?

A: Heroin…Honestly, I want to go into a rehabilitation center…I was there a while back, but I didn't last long…

Q: How's the program? Does it use Methadone?

A: No, you just have to withstand the withdrawal symptoms. The time that I was there I wasn't given any medication.

[Sabina, Age 45]

## Discussion

In this study, sex workers with a history of sexual exploitation or youth sex work provided recommendations to reduce susceptibility to sexual exploitation by offering young women and migrants appropriate social supports and peer-based education. They also emphasized the need to mitigate sex industry-related harms – via improved access to HIV prevention resources and a strengthened response to gender-based violence – and stressed the need to provide sex workers who wish to exit the sex industry with viable exit opportunities (e.g., vocational trainings/placements, effective drug treatment).

*Reduce susceptibility to exploitation:* Our study findings highlight the critical need for structural interventions to reduce vulnerability to exploitation, especially within higher-risk border contexts. Current approaches to prevent sex trafficking have often deflected attention away from the broader contextual factors that give rise to exploitation and, instead, often focus on the criminalization of women and girls involved in the sex industry [56]. In this study, participants offered their own alternative suggestions for approaches to prevent sexual exploitation and address the vulnerabilities experienced by youth in sex work, such as peer-based outreach. Peer-based approaches have been shown to promote condom use among establishment-based sex workers in the Philippines [57]. Our findings build on this work by suggesting that such interventions may be amenable to adaptation for the purpose of preventing sexual exploitation and HIV infection in Mexico. More broadly, our data add to a growing body of evidence indicating the critical need to shift towards interventions addressing the root causes that facilitate exploitation [11], [58], [59]. Public policies that provide social supports aimed at reducing to the vulnerabilities experienced by unaccompanied and marginalized migrant women and girls are especially needed in Mexico [37], [60].

*Mitigate harms:* In this study, participants illustrated a clear need for psychosocial services to address past and ongoing experiences of trauma. This indicates an urgent need for evidence-based mental health care tailored to meet the unique circumstances of survivors of sexual exploitation in Mexico, echoing previous studies among women and girls accessing post-trafficking services in Europe [61]. Our findings are supported by previous studies indicating close linkages between past and current victimization and trauma [62]. For instance, a substantial body of literature indicates that trauma (e.g., sexual and physical violence) may relate to an increased likelihood of sex industry involvement [63]–[68] and subsequent HIV risks [62], [68]–[72]. Future scholarship and program development aimed at the design, testing, and implementation of trauma programs are recommended to promote HIV prevention and the broader wellbeing of sex workers who have experienced prior exploitation. The close involvement of those involved in the sex industry in the creation and implementation of mental health and psychosocial services is recommended, such as through an advisory board of trafficking survivors and current sex workers.

Further, the findings of this study shed light on the need for structural reforms to increase transparency and strengthen the response to gender-based violence among marginalized women and girls (e.g., independent human rights monitoring of law enforcement actions). Such actions are critical considering the disturbingly high levels of violence, abusive police practices and institutional barriers to reporting violence (e.g., threats, intimidation, corruption) described in this setting and other international contexts [3], [11], [61], [73]–[79]. For example, between 50–100 percent of street-based sex workers report physical, sexual and economic violence [80]–[84], which has been shown to compromise women's' abilities to refuse unsafe sex and is associated with HIV infection [81], [84]–[86].

*Provide viable sex industry exit opportunities:* Our finding regarding participants' desires for economic alternatives to sex industry involvement is supported by research with sex workers in Mexico and other settings [87]–[89]. In a qualitative study in China, approximately half of women were lured into sex work and initially deceived regarding their working conditions; consequently, they almost unanimously reported dissatisfaction with their working conditions and a desire to leave the sex industry [87]. Among current and former sex workers in Thailand, those who had successfully exited the sex industry highlighted the importance of receiving appropriate support to facilitate their transition out of sex work, while those who remained in sex work often did so due to economic factors and other structural factors such as by low education levels [88]. Micro-enterprise programs represent one potentially viable means of supporting alternative opportunities and enabling HIV prevention among women who desire to leave or reduce their reliance on sex work. In Kenya, among sex workers participating in a micro-enterprise intervention, two-thirds had operational businesses by the end of the study, nearly half stopped sex work, and consistent condom use with regular partners increased by 18.5% [90]. However, it is imperative for such interventions to be underpinned by a rights-based approach to promoting the health and well-being of all those in the sex industry, rather than as a rescue attempt [86]. Future research in this area should explore the potential role of microfinance as a potential means of offering economic alternatives to women and girls who may desire them in Mexico and internationally.

Moreover, women's recommendations to improve access to effective drug treatment are in line with prior research documenting the linkages between failed efforts to abstain from drug use and youth sex work initiation [10]. In Mexico and other international settings, having sought but been unable to access addiction treatment has been associated with sex work involvement, including youth sex work entry [10] [91]. Such linkages may be explained by the concerns women shared regarding mistreatment and human rights abuses in drug rehabilitation centers, which echo previous studies which draw attention to the critical need for resources to strengthen drug treatment program quality in Mexico [92]. While Mexico is currently undertaking steps to regulate and improve the quality of its drug treatment programs [93], the evaluation and reform of drug treatment programs with regards to such abuses is recommended as part of this wider initiative.

Across study themes, women's narratives indicated the complexity of reasons for beginning and continuing to exchange sex, suggesting that experiences of sexual exploitation and youth sex work entry are far more nuanced than current conceptualizations and definitions reflect [60], [94]. In light of the fact that many of those who involuntarily entered the sex industry continued to exchange sex in the absence of coercion, non-victimizing, community-based interventions that recognize this and build upon women's resilience and skills in a sensitive, informed manner are recommended.

### Strengths and limitations

Whereas academic studies and media reports often rely on indirect sources to gather data on sexual exploitation (e.g., service provider records, key informants), this study gathered the perspectives of women with a history of sexual exploitation or youth sex work, which is responsive to calls for a response to sex trafficking that addresses its root causes and is based in sex workers' and trafficking survivor's experiences [58]. This approach enabled us to identify the health and social needs that participants perceived as most pressing, and yielded rich and insightful data. For example, our findings highlighted women's willingness to build upon their own experience to reduce sexual exploitation among their peers, underscoring the potential for future peer-based interventions. By incorporating the target population's input and priorities, future interventions may be more effective, sustainable, and meaningful to the communities they are meant to serve.

### Conclusions

In this study, we aimed to gather recommendations for ways to reduce susceptibility to sexual exploitation and HIV infection from women with a history of sexual exploitation or youth sex work. Women's main suggestions were to develop interventions that aimed to: (1) reduce susceptibility to sexual exploitation by providing social support and peer-based education; (2) mitigate harms by improving access to HIV prevention resources and psychological support, and reducing gender-based violence; and (3) provide opportunities to exit the sex industry via vocational trainings/placements and improved access to effective drug treatment.

In conjunction with previous research indicating the pervasive impacts of violence, trauma, and limited economic opportunities on risk of HIV and susceptibility to sexual exploitation [3], [45], these data highlight the critical need for interventions addressing structural risks (e.g., poverty, gender-based inequalities and violence) among vulnerable women and girls. Findings suggest the need to develop and test interventions to reduce vulnerability to sexual exploitation, mitigate harm and provide sex industry exit opportunities for those who may desire them, both in Mexico and other similar settings.
